# The Gut-liver Axis in Immune Remodeling: New insight into Liver Diseases

**DOI:** 10.7150/ijbs.46405

**Published:** 2020-06-23

**Authors:** Xinyu Yang, Di Lu, Jianyong Zhuo, Zuyuan Lin, Modan Yang, Xiao Xu

**Affiliations:** 1 Department of Hepatobiliary and Pancreatic Surgery, the First Affiliated Hospital, Zhejiang University School of Medicine, 79 Qingchun Road, Hangzhou 310003, China.; 2 NHFPC Key Laboratory of Combined Multi-Organ Transplantation, Hangzhou, 310003, China.

**Keywords:** gut-liver axis, immunity, gut microbiome, dysbiosis, immunotherapy

## Abstract

The gut microbiota consists of a dynamic multispecies community of bacteria, fungi, archaea, and protozoans, playing a fundamental role in the induction, training, and function of the host immune system. The liver is anatomically and physiologically linked to the gut microbiota via enterohepatic circulation, specifically receiving intestine-derived blood through the portal vein. The gut microbiota is crucial for maintaining immune homeostasis of the gut-liver axis. A shift in gut microbiota composition can result in activation of the mucosal immune response causing homeostasis imbalance. This imbalance results in translocation of bacteria and migration of immune cells to the liver, which is related to inflammation-mediated liver injury and tumor progression. In this review, we outline the role of the gut microbiota in modulating host immunity and summarize novel findings and recent advances in immune-based therapeutics associated with the gut-liver axis. Moving forward, a deep understanding of the microbiome-immune-liver axis will provide insight into the basic mechanisms of gut microbiota dysbiosis affecting liver diseases.

## Introduction

The reciprocal interaction of the gut-liver axis is established through the vascular route of the portal vein that directly transports gut-derived products to the liver, and the liver feedback route by which bile and antibodies travel to the intestine 1. The intestinal barrier, a functional and anatomical structure consisting of intestinal mucosa and vascular endothelium, acts as a playground for the connections between the gut and the liver.

As an important constituent of the mucosal immune system, gut-associated lymphoid tissue (GALT) constructs a local immune environment that is both defensive and tolerant. The liver, as an organ linked to GALT, contributes to immune surveillance. 2 The liver, particularly enriched in innate immune cells, is a central immunological organ with high exposure to circulating antigens and endotoxins from the gut microbiota. 3 The dysregulation of the gut and liver immune system is involved with intestinal and liver diseases. 2 The intestinal mucosal surface forms a biophysical barrier, and mucus may enhance homeostasis by inducing immunoregulatory signals. For instance, MUC2 mucin has been found to imprint dendritic cells (DC)s tolerance after direct uptake 4, 5. Intestinal epithelial cells (IECs) secrete conditioning cytokines, including thymic stromal lymphopoietin (TSLP) and transforming growth factor-β (TGF-β) as well as prostaglandins (PGs) to prime DCs to promote the induction of T helper cell 17 (Th17) differentiation 6-10. In addition, IECs also exert a strong influence on local IgA response by producing factors such as B-cell activating factor (BAFF, also known as TNFSF13B) and a proliferation-inducing ligand (APRIL, also known as TNFSF13) 11. In the lamina propria (LP), beneath IECs, both DCs and macrophages have specific adaptations promoting tolerance through the control of regulatory T cells (Tregs) and IgA+ B cells, which contribute to tolerance by displaying key gut-homing receptors CCR9 and α4β7 12. However, upon shifting to inflammation, T helper 1 (Th1) and Th17 responses are induced. Meanwhile, in the liver, the inflammatory activation of hepatic stellate and Kupffer cells can recruit innate immune effectors, including neutrophils, monocytes, natural killer (NK) cells and natural killer T (NKT) cells 3. In addition, enterohepatic circulation of bile and blood carries products of digestion, along with immune molecules, antigens and microbial products; can also modulate intestinal immunity to some extent 13-15.

Here, a comprehensive review was conducted to illustrate the crosstalk between the gut microbiome and the host innate and adaptive immunity, highlighting the impact of gut microbiota dysbiosis on systematic immunity. In particular, the gut-liver axis involving the intestinal microbiome and hepatic immune system was outlined as a novel paradigm in immune-based therapies, on the basis of its vital role in the immune response.

## The crosstalk between the microbiome and immunity

The immune system acts as a bridge to maintain the symbiotic relationship between the microbiome and the host. The gut microbiota modulates the host immune system to some extent, and the immune system inversely influences the composition of the gut microbiota. At the same time, owing to major changes induced by bacterial colonization of the intestinal tract 16, it is thought that the mucosal immune system is different from the systemic immune system, and is highly specialized and defined. In particular, it is thought that the mucosal immune system maintains gut homeostasis by promoting a beneficial microbiota composition, limiting the development of pathological processes and restricting microbial overgrowth 17.

The intestinal mucosa has a single cell layer of epithelial cells that separates the gut lumen harboring the commensal bacterial and foodborne pathogens from the body. In the mucosa-associated lymphoid tissue (MALT), GALT is composed of Peyer's patches (PPs) and various immune cells, such as antigen-presenting cells (APCs), innate lymphoid cells, and T and B cells. The GALT serves as an essential component of the immune system and plays a critical role in systemic and local immune responses.

### Microbial immunomodulation in systemic immunity

The microbiota actively shapes the host systemic immune response by mediating immune cell priming. DCs migrate to mesenteric lymph nodes (mLNs), where they present antigens to stimulate the production of Treg cells and effector T cells. These cells can balance gut tolerance and immunity by transmitting signals to the whole body, such as the production of regulatory cytokines (TGF-β, IL-10, and IL-35), and exerting the appropriate immunological reaction to combat specific pathogens by cross-reacting with similar epitopes 18-20. The balance of beneficial bacteria versus pathogenic bacteria is referred to as “eubiosis”, and is important in maintaining immunity. In contrast, when dysbiosis occurs (due to various causes, e.g., poor colonization, antibodies treatment or an unbalanced, unhealthy diet), the microbiota loses its anti-infectious potency against pathogenic bacteria. In addition, alterations in the microbiota under the new condition of dysbiosis can lead to a pathogenic tendency by producing opportunistic infections. For example, the induced alteration of the intestinal microbiota after antibiotic use could lead to metabolic disturbances, and therefore increase susceptibility to infections (e.g., fungal and *Clostridium difficile* infections) 21. Gut dysbiosis may lead to a number of diseases, including gastrointestinal disorders, obesity, cardiovascular diseases, allergies and central nervous system-related diseases, through a series of alterations 22-24. This alteration involves the disruption of the mucosal barrier, which impairs local immune responses. Given this intestinal dysbiosis, translocated bacteria and their derived products enter the peripheral circulation, thus influencing the systemic immunity through activating TLR signaling pathways and subsequently triggering a cascade of inflammatory cytokines 25. During this process, the release of pro-inflammatory cytokines increases, changing the cytokine environment in the intestinal mucosa and mLNs into an inflammatory phenotype. Eventually, a deep inflammatory state is induced throughout the body 26.

### The microbiota in gut immunomodulation

Local immunity is facilitated by pathogen recognition receptors (PRRs)-mediated recognition of pathogen-associated molecular patterns (PAMPs). PRRs, including TLRs on IECs and innate immune effectors in the gut, are a class of germline-encoded receptors that recognize PAMPs. The activation of PRRs is crucial for the initiation of innate immunity, which plays a key role in the first-line of defense until more specific adaptive immunity is developed.

Furthermore, microbiota-derived metabolites, including short-chain fatty acids (SCFAs), can also modulate local immune responses. SCFAs exert strong epigenetic regulatory effects on B cell differentiation by promoting the production of both IgA and IgG isotypes 27-29. SCFAs can also upregulate three important metabolic processes, glycolysis, oxidative phosphorylation and lipogenesis in B‐cells, which are necessary to produce cellular building blocks and energy to support plasma B‐cell differentiation 30. mLNs are sites in which commensal bacteria transform adaptive immune responses, mainly by promoting the differentiation of naive T cells 31. Once DCs become mature, they migrate to mLNs, transforming naive T cells into CD4^+^ Tregs and Th17 cells, which possess the ability of modulating intestinal immune balance 32, 33. Tregs have the ability to induce mucosal tolerance and produce of immunosuppressive cytokines (e.g., IL-10). Of note, continuous crosstalk occurs between intestinal symbionts and mucosal T cells (e.g., Tregs) because bacterial metabolites such as SCFAs promote the maintenance of T cells in the intestine. The function of SCFAs relies on their capacity to suppress histone deacetylase (HDAC) activity, indicating the presence of epigenetic regulation 34. In detail, the major components of SCFAs, including propionate and butyrate, can inhibit the HDAC 1 and 3. HDACs and histone acetylase (HATs) induce the histone acetylation, which is critically important epigenetic mechanisms involved in the regulation of gene expression by serving as a switch between permissive (via HAT-induced acetylation) and repressive chromatin (through HDAC-driven deacetylation). Butyrate also seems to influence PAMPs-induced inflammatory state, a previous study has found that butyrate inhibits peptidoglycan-induced TNF-α and IL-1β expression in THP-1 cells 35.

Resident microbiota contributes to the coordination of Treg/Th17 axis and safeguarding the mucosa. Microbiota dependent TLR signaling is involved in the regulation of inflammation and tolerance. TLR2/MyD88signaling is required for generation and expansion of Nrp1^low^ Foxp3^+^ Tregs and Treg17 cells in oral and gut mucosa. 36 The capsular polysaccharide A of the *Bacteroides fragilis* can promote the production of IL-10 by Foxp3^+^ Tregs in a TLR2 dependent manner, thus facilitating the mucosal tolerance 37. The presence of commensal bacteria is required for the induction of steady-state Th17 cells in the intestinal lamina propria. In germ-free (GF) mice, Th17 cells are significantly decreased, but they can be induced by segmented filamentous bacteria 38, 39. Th17 cells in the lamina propria of the gut play a critical role in preventing pathogen infection. Modulation of Th17 cells is currently viewed as a potentially pharmacological target. Inhibition of a Th17 response would result in downregulation of pro-inflammatory IL-17 production 40.

Furthermore, Th2 immune responses contribute to the maintenance of mucosal homeostasis through increased secretion of IL-4, IL-5, IL-9, IL-13 and IL-21, which confer protection against helminthic infection 41. The “core” signature of Th2 responses is the secretion of the cytokines IL-4, IL-5, and IL-13 by lymphocytes that express transcription factors, such as GATA binding protein-3, STAT-5, and STAT-6 42. A healthy balance of Th1/Th2 cells is essential for immune regulation. The gut microbiota and its metabolites influence the balance of Th1/Th2 cells ratio in the intestinal tract. Colonization of GF mice with *Bacteroides fragilis* was found to be sufficient to correct an imbalance between Th1 and Th2 cells 43. Recent research has also indicated that yeast β-glucan, a polysaccharide of the gut microbiota, can contribute to the differentiation and secretion of Th2 cells by elevating the expression of GATA3 mRNA 44.

The interaction between microbiota and immunity also depend on the physiological location. With an oxidative stress sensitive (Ox-S)/oxidative stress-resistant (Ox-R) bacterial ratio increase, the colonic microbiota-immunity interaction is different from that in the small intestine in terms of oxygen tolerance 45. Production of SCFAs, especially butyrate, in the gut microbiome is required for maturation of the gut microbiota 46. Butyrate is produced from acetate and lactate by the Ox-S gut microbiota, mainly *Lachnospiraceae, Ruminococcaceae and Bacteroidetes* 47. In microbiota-immunity interactions, on the one hand, butyrate downregulates gut mucosal immunity with an increase in FoxP3^+^ Tregs in the colon. On the other hand, butyrate upregulates antigen-specific immune-response induction through decreased NKp46 group 3 innate lymphoid cells (ILC3s) in the Peyer's patches of the terminal ileum 48, 49. Therefore, butyrate modulates gut mucosal immunity depending on the physiological location, with induction of antigen-specific immune responses in terminal ileal PPs, but immunological tolerance within the colon. Different immune regulations between the terminal ileum and the colon play a vital role in immunosurveillance and anaerobic biological processes for host health.

### The gut microbiota in shaping hepatic immunity

**Figure 1:**
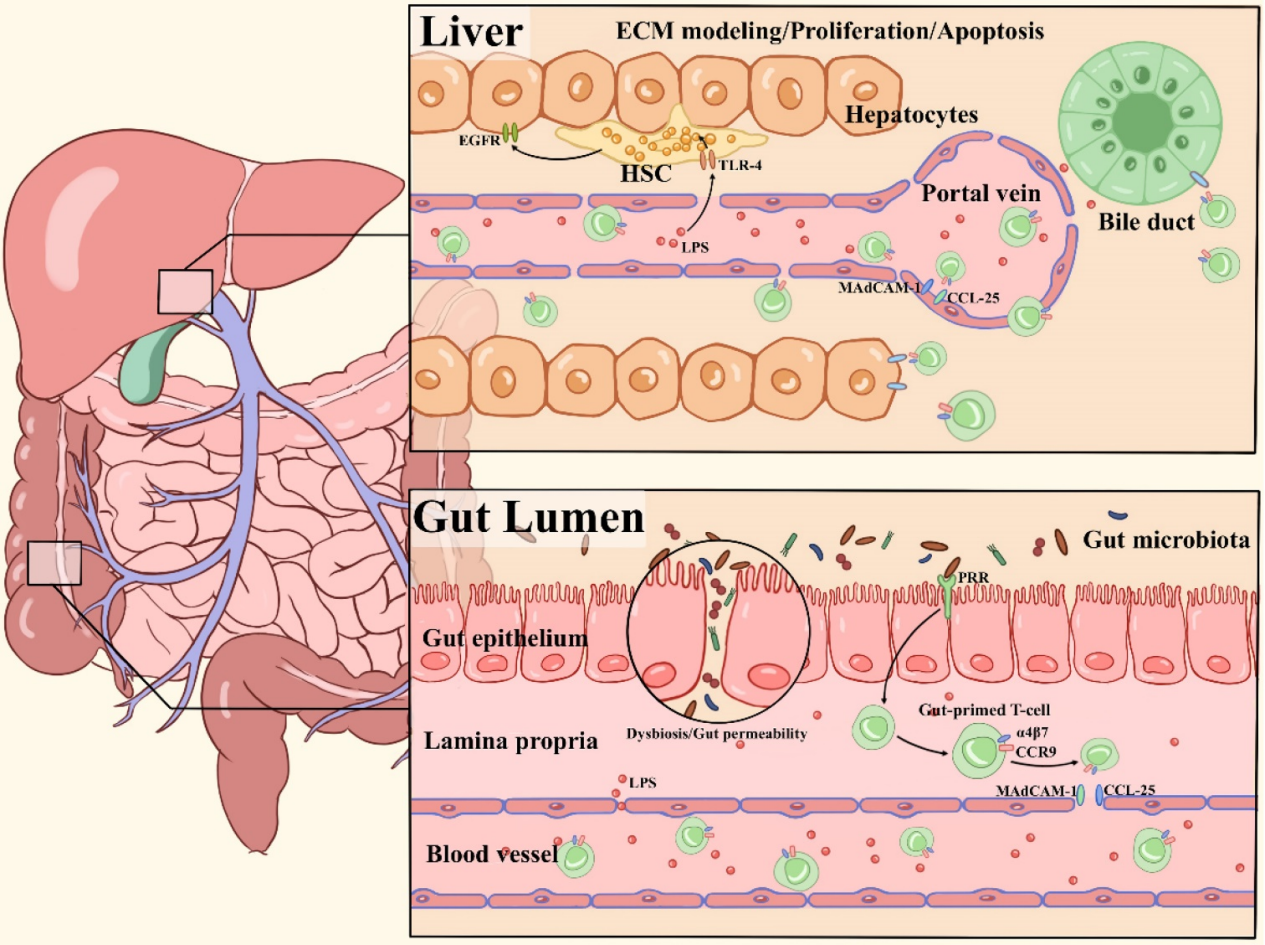
** Overview of major pathways of the gut microbiota in shaping hepatic immunity.** (1) Chronic inflammatory liver diseases are associated with gut microbiota dysbiosis, intestinal permeability changes, and MAMPs (LPS) translocation to the liver. TLR4 signaling is activated by LPS in hepatic stellate cells (HSCs) and hepatocytes, leading to extracellular matrix (ECM) remodeling, secretion of proinflammatory cytokines and activation of epidermal growth factor (EGF) family members, which influence the proliferation and apoptosis of hepatocytes. (2) Pathogens derived from bacterial translocation from the inflamed gut to the portal circulation due to increased intestinal permeability, driving the local inflammation via PRR activation. The naive T cells are imprinted with the gut-homing receptors integrin α4β7 and CC-chemokine receptor 9 (CCR9), these so-called gut-primed T cells will recirculate into the liver via venules by binding to CCL25 and MAdCAM-1 on hepatic endothelial cells. Then, chemokines secreted by epithelial target cells (hepatocytes or biliary epithelial cell) are in response to the activation of chemokine receptors such as CCR6 on effector cells. As a result, chronic inflammation, immune attack and destruction of bile ducts occur.

The liver is continuously exposed to an overload of antigenic stimuli which includes pathogens and endotoxins from the gut microbiota, and plays a critical role in maintaining immunological tolerance 50-52. The liver is considered a unique immunological organ with a predominantly innate immune role, as it contains an unusually large number of innate immune cells, including NK cells, NKT cells, macrophages and γδ T cells 53. A previous report demonstrated the inflammasome-IL-18 regulatory signaling circuit impacted maturation of hepatic NK cells, surface expression of the death ligand FasL, and capacity to kill FasL sensitive tumors. This study defines a regulatory circuitry in the innate immune system that links microbiota-derived Nlrp3 inflammasome activation by endogenous IL-18 signal to effective hepatic NK cell-mediated tumor attack 54. The microbiota also sustains the hepatic IL-17A-producing γδT (γδT-17) cell homeostasis, including activation, survival and proliferation. Li et al. showed that colonization with *E. coli* induces generation of γδT-17 cells in a dose-dependent manner 55. Gut bacteria shed microbial-associated molecule patterns (MAMPs), such as lipopolysaccharide (LPS) and endotoxin, into the portal venous circulation. The molecules can affect the Kupffer cell phenotype through TLR ligands and trigger a subsequent adaptive immune response via Kupffer cell-derived pro-inflammatory cytokines, thus shaping liver immunity 56, 57. Here, we discuss in detail two main pathways by which the gut microbiota shapes hepatic immune cell responses to modulate liver-associated diseases (**Figure 1**).

#### Microbial translocation and immune activation in the liver

The intestinal mucosal immune system is comprised of specialized structures and sites, including PPs, lymph nodes, the lamina propria and the epithelium, that contain a variety of cells that participate in continuous activation, migration and homing 58. Among them, the gut-draining mLNs are critical sites for orchestrating adaptive immunity. To support the failing intestinal barrier, the liver acts as a second firewall, filtering bacteria that drain from the intestine into the hepatic portal vein 59, 60. Pattern recognition receptors (PRRs) function as sensors of MAMPs, such as LPS, lipoteichoic acid (LTA), peptidoglycan and lipoproteins 61. Once MAMPs arrive to the liver through the portal vein, they can activate innate immune cells expressing PRRs, including Kupffer cells, hepatic sinusoidal endothelial cells (HSECs) and biliary epithelial cells, via PRRs binding (e.g., Toll-like receptor 4) and induce inflammation 62-64. For example, the LPS/TLR4 pathway upregulates the epiregulin hepatomitogen, an epidermal growth factor (EGF) family member in HSECs, leading to EGFR and HER2 activation, whereas it inhibits hepatocyte apoptosis during the late stages of hepatocarcinogenesis 65, 66. Increased gut-derived MAMPs shift to the liver during dysbiosis and subsequently shape the hepatic immune milieu by regulating inflammatory cytokines. LPS/TLR4 activation in Kupffer cells induces the secretion of pro-inflammatory cytokines, such as TNF-α and IL-6. These elevated cytokines enhance the permeability of the hepatic sinus and the proliferation of hepatocytes, resulting in increased aggressiveness of hepatocellular carcinoma (HCC) 67. In addition, as IL-6 is an activator of the JAK-STAT signaling pathway, its upregulation can lead to the polarization of M2 macrophages, potentially contributing to HCC metastasis and drug resistance in chemotherapy 68. In summary, bacterial translocation might drive excessive immune responses that may compromise the health of the host. Liver cells, especially hepatocytes and cholangiocytes, are particularly susceptible to changes in the immune milieu. The 'leaky gut' hypothesis also links translocating gut microbial products with the onset and progression of nonalcoholic fatty liver disease (NAFLD) and alcohol-related liver disease (ALD), and for a long time, they were considered one of its major contributors. Compared with healthy individuals, patients with NAFLD were shown to have increased intestinal permeability and tight junction alterations 69. In addition, chronic alcohol abuse results in a disruption of the intestinal barrier, related to the development and progression of ALD 70.

#### Recruitment of mucosal lymphocytes into the liver

In parallel to the 'leaky gut' hypothesis, a 'gut lymphocyte homing' hypothesis has been adopted. It studies the reciprocal interaction between the mucosal immune system and hepatic immunity through the gut-liver axis. The adhesion of lymphocytes into the liver differs from the classical migration pathway as described earlier 71, 72. Among them, there is an important role for lymphocyte adhesion molecules expressed by HSECs, including vascular cell adhesion molecule-1 (VCAM-1), intercellular adhesion molecule-1 (ICAM-1) and common lymphatic endothelial and vascular endothelial receptor-1 (CLEVER-1) 73. Hepatic VCAM-1 is only weakly expressed on human portal ECs, but its expression is increased by inflammatory cytokines, and it therefore contributes to homing through both the portal veins and hepatic sinusoids 74, 75. Interestingly, in an antigen-driven mouse model of biliary injury, VCAM-1-mediated adhesion of α4β1-positive hepatic T cells to cholangiocytes reduced apoptosis, thus promoting T cell survival and continuance of hepatic inflammation 76. The hepatic endothelium has been shown to aberrantly express the gut-specific chemokine CCL25 and recruit gut-homing CCR9^+^ lymphocytes by binding to mucosal addressin cell adhesion molecule-1 (MAdCAM-1) 77. These results indicate that gut-primed T cells migrate from the gut to the liver and induce immune responses in the liver 78. The pathogenesis of primary sclerosing cholangitis (PSC) has been suggested to be related to inflammatory bowel disease (IBD) and inflammation in the portal tract. The 'leaky gut' and 'gut lymphocyte homing' hypothesis explain the correlation observed between IBD and PSC 79. These phenomenons highlight the association of the gut-liver axis in these immune disorders.

### The microbiota-immune interaction in liver diseases

Since the portal vein provides approximately 70% of the liver's blood supply, dysbiosis of the gut microbiota can therefore shape immune cell responses in the liver and is related to various liver diseases including NAFLD and ALD 80-85. Salzman et al. believed that the negative effects of gut dysbiosis are accompanied by gut microbiota-mediated inflammation of the local mucosa that encourages mucosal immune dysfunction, thus contributing to NAFLD pathogenesis 86. It is documented that certain gut microbiota members, including members of the *Bifidobacterium* genus, influence Treg development, whereas others, such as segmented filamentous bacteria (SFB), promote Th17 development 87-89. These particular members of the microbiota are associated with liver diseases, along with immune-related biological processes, including activation of innate and adaptive immune responses, suppression of inflammatory cytokine production and inhibition of immune cell recruitment 90.

## The immune shift under the treatment of liver diseases: the role of the gut-liver axis

Mounting evidence highlights the role of the commensal microbiome in influencing the immune milieu of liver diseases, which, in turn, suggests the potential therapeutic utility of regulating the immune response via multiple microbiome manipulation methods, such as antibiotics, probiotics, prebiotics, fecal microbiome transplant (FMT), diet regulation, and administration of bacterial consortia. Efforts are currently underway to produce or enhance therapeutic responses by influencing the immune milieu-associated with the gut-liver axis. Several studies have presented a preliminary benefit in malignant and nonmalignant liver diseases.

### The gut microbiota and autoimmune liver diseases

PSC is the most common autoimmune liver disease, characterized by a progressive immune-mediated liver damage that leads to fibrosis of the biliary tree with chronic cholestasis and often end-stage liver cirrhosis 91. PSC is often associated with IBD, because colonic inflammation can lead to increased intestinal epithelium permeability and bacterial translocation to the liver, accelerating activated T cell migration from the intestine to the liver triggering immune-mediated damage 92. Recently, a study has shed light on this association, showing that *Klebsiella pneumoniae (K. pneumoniae)* can disrupt gut barrier integrity and then trigger innate immune responses in the liver following translocation 93. Using gnotobiotic mice and bacterial-organoid co-culture system, researchers demonstrated that PSC-derived *K. pneumoniae* was related to bacterial translocation and susceptibility to Th17-mediated hepatobiliary injuries. These results indicate that disease-specific bacteria might serve as a potential therapeutic target for PSC.

### The gut microbiota and cancer immunotherapy

Several studies have reported that immunotherapy responders have differential gut microbiota signatures than nonresponders, and these specific signatures are related to enhanced systemic immunity and increased intratumoral immune infiltration. Recently, reports found that responder and nonresponder phenotypes could be replicated in antibiotic-treated or germ-free mouse models through fecal microbiota transplant (FMT). This phenomenon implied that therapeutic responses can be regulated through the modulation of the gut microbiota 94-97. As shown in **Table 1**, some clinical trials were also conducted to explore the value of the gut microbiota in improving immunotherapy effects. In addition, a study reported that cyclophosphamide (an immunostimulatory agent) alters the composition of the natural microbiota in the small intestine of mice and facilitates a shift of selected Gram-positive bacteria to secondary lymphoid organs. These bacteria stimulate the generation of a specific subset of “pathogenic” Th17 cells and the memory Th1 cell immune response. These cells enhance the therapeutic effect of cyclophosphamide by expressing IL-17. This conversion into IL-17-producing cells was not observed in the absence of the gut microbiota 98. The efficacy of immune checkpoint inhibitors (ICIs), including anti-PD-1/PD-L1 and anti-CTLA-4 agents, may be affected by the components of the gut microbiota. A recent study found that bacterial species, including *Bifidobacterium longum, Collinsella aerofaciens,* and* Enterococcus faecium*, were more abundant in anti-PD-L1 therapy responders than in the nonresponders. Remodeling the gut microbiota of germ-free mice with fecal material obtained from patients who responded to anti-PD-L1 agents could enhance T cell responses and improve the efficacy of anti-PD-L1 therapy 97. Hepatocellular carcinoma (HCC) and cholangiocarcinoma (CCA) are the most common histological types of liver cancer. The gut microbiota is also associated with the response to anti-PD-1 immunotherapy in HCC patients. Through metagenomic sequencing, a study reported that fecal samples from HCC patients responding to anti-PD-1 immunotherapy showed higher taxa richness and more gene counts than those from nonresponders. These results highlight an important role of microbiota in disease monitoring and treatment decision-making 99. Fundamental research also revealed that microbiome-induced innate immune change has an impact on the antitumor immune response in liver tumors. Using mouse models of primary liver tumors and metastatic liver tumors, Ma and colleagues found that *Clostridium* species could inhibit the recruitment of hepatic NKT cells and thereafter suppress antitumor immunity in the liver, against both primary and metastatic liver tumors. In addition, antibiotic treatment was shown to alter the composition of the gut microbiota and inhibit tumor growth 100. In a summary, these treatment-responsive microbiome signatures suggest high potential for identifying novel combinations with checkpoint inhibitors. However, numerous issues remain to be addressed regarding the microbial product administered.

### The impact of gut dysbiosis on ischemia reperfusion injury (IRI) and immune-mediated allograft injury

Both animal and human studies have revealed that the gut-liver axis acts as an important modulator in allograft innate and adaptive immunity, implicating the therapeutic effect of microbiota-based treatment in immune-mediated allograft injury 101, 102. IRI causes organ dysfunction and failure after liver surgery and represents a major risk factor for the development of both acute and chronic graft rejection in liver transplantation (LT) 103. Importantly, it is the limiting factor in the utility of marginal or extended criteria donor organs, which are highly susceptible to IRI, contributing to severe organ shortages. IRI is a dynamic process in which innate and adaptive immune inflammatory responses play an essential role in developing early allograft dysfunction (EAD) or primary nonfunction (PNF) 104, 105. The impact of the gut microbiota on early innate immune activation during IRI has been reported. A protective effect was found in a mouse IRI model by administrating probiotics, mainly *Bifidobacterium* and *Lactobacillus* 106. Furthermore, in a rat model of LT, liver ischemic preconditioning not only improved hepatic graft function and intestinal barrier function, but also promoted restoration of the intestinal microbiota following LT, especially increasing *Lactobacillus, Bifidobacterium,* and* Clostridiales*. This process may further benefit hepatic grafts via positive feedback of the gut-liver axis 107. Microbiota-derived SCFAs produced by the fermentation of nondigestible fiber can enable communications between the microbiome and host tissues and act as critical modulators in liver immune homeostasis. Intravenous administration of butyrate, a four-carbon fatty acid, can alleviate IRI-induced liver injury by suppressing inflammatory factor production and preventing NF-κB activation in Kupffer cells 104, 108.

### The gut microbiota in other liver diseases - emerging indications

The gut microbiota is also involved in the immunoregulation of viral, alcoholic and drug-related liver diseases. In chronic hepatitis B virus (HBV) infection, other than being directly caused by the adaptive immune response, liver injury is also indirectly caused by the innate immune response through gut microbiota-produced PAMPs 109. TLRs are the main pattern recognition receptors in the innate immune system and play a vital role in the immune response 110. The gut microbiota plays critical roles in drug metabolism. Individual variations in the gut microbiota contribute to the interindividual differences in response to drug therapy, including differences in drug-induced toxicity and efficacy 111. Microbiota-derived metabolites can indirectly affect xenobiotic metabolism pathways. A previous study found that the relative abundance of *Mucispirillum, Turicibacter* and *Ruminococcus* before acetaminophen (APAP) dosing was correlated with increased hepatotoxicity, indicating APAP-induced acute liver injury 112. In addition, gut dysbiosis is also associated with immune dysregulation during the onset and progression of alcohol-related liver disease (ALD). Researchers found that intestinal deficiency in two antimicrobial proteins, regenerating islet derived (Reg)-3b and Reg3g, can promote the progression of ethanol-induced fatty liver disease toward steatohepatitis 113, 114. The intestinal mucus layer is composed of mucins, predominantly MUC2, secreted by goblet cells of the intestine. Muc2^-/-^ mice are protected from intestinal bacterial overgrowth and dysbiosis in response to alcohol feeding 115. *Enterococcus faecalis* (*E. faecalis*) is related with the progression of ALD, and a recent study indicated that bacteriophages can attenuate ALD through specifically targeting the cytolytic *E. faecalis* 116, 117.

## Conclusions and future trends

The gut microbiota is a central participant in regulating hepatic immunity through the gut-liver axis, which refers to the reciprocal interaction that takes place between both the gut and its microbiota, and the gut and the liver on the other. Furthermore, there is growing evidence that dysregulation of gut-liver immunity leads to the progression of liver diseases, including malignant tumors. Thus, the mechanisms by which innate and adaptive immunity are influenced through the gut-liver axis have become attractive research topics.

Elucidation of the detailed immune changes associated with the gut microbiota induced by the gut-liver axis may contribute to the development of promising therapeutic strategies for liver diseases. A liver cancer-specific gut microbiota and the immune response induced by gut microbial species might be uncovered in the near future. Likewise, microbial-based interventions have demonstrated a benefit in improving allograft function and reducing the risk of post-LT complications, implicating that microbiota-based therapies will be utilized widely to improve clinical outcomes in post-LT patients. Currently, preclinical studies have demonstrated the bidirectional effect of the gut microbiota on the response to immunotherapy in mouse models. However, the biological mechanisms at cellular and molecular levels underlying the relation between gut microbiota and positive response to ICIs need to be further elucidated. In addition, whether the gut microbiome as a whole, or specific bacteria, can influence therapeutic responsiveness, and which specific composition is the most ideal for promoting cancer immunotherapy are still unclear. Therefore, thoroughly studying the multiplicity of therapeutic options, such as diet modification and FMT, is required in future clinical research. With 16S rRNA gene-based microbial profiling technology, we can better identify the composition of the gut microbiota. Moreover, a comprehensive characterization at the species level can further promote our understanding of the effects of the gut microbiome on gut-liver immunity, thus allowing microbiome modulation to enhance the efficacy of immunotherapy in liver diseases. Moreover, advanced approaches such as clustered regularly interspaced short palindromic repeat (CRISPR)-based technologies have revolutionized the genome editing field and have already been applied to the development of novel antimicrobial strategies 118, 119. With specific genetic properties, selective and efficient eradication of pathogenic bacteria has become a reality. Though still rather early in clinical application, these emerging technologies indicate exciting possibilities for microbiota modulation in the future.

In conclusion, it is still unclear which specific composition of the gut microbiome is most conducive to promoting a beneficial immune response. There are a variety of treatments that alter the microbiome, which require a future careful testing in the setting of clinical trials. Only by fully understanding these interactions we can learn to optimally target the microbiota to prevent and alleviate liver diseases via the remodeling of the immune milieu between the gut and the liver.

## Figures and Tables

**Table 1:** Clinical trials aiming to improve cancer immunotherapy by modulating the gut microbiota

Registration number	Cancer type	n	Objective	Intervention	Outcome measure(s)	Country
NCT03358511	Breast cancer	20	To assess the impact of presurgical probiotics on antitumor immune function	Primal Defense Ultra® probiotic formula.	Mean number of cytotoxic T lymphocytes (CD8^+^ cells).	USA
NCT03772899	Melanoma	20	To assess the safety of combining FMT and immunotherapy in advanced melanoma patients	FMT combined with approved immunotherapy (either pembrolizumab or nivolumab).	Adverse effect assessments.	Canada
NCT04130763	Gastrointestinal system cancer	5	To study the use of FMT in patients with gastrointestinal system cancer for whom anti-PD-1 treatment failed	FMT capsule produced by the gut microbiota of these healthy people.	ORR; the safety of FMT capsule was assessed by adverse events.	China
NCT03341143	Melanoma	20	To study the concurrent use of FMT with pembrolizumab in patients with anti-PD-1 agent-resistant/refractory melanoma	FMT combined with Pembrolizumab.	ORR; alterations in T cell composition and function; alterations in innate/adaptive immune system subsets.	USA
